# Specific IgE test: sex, age and pathologies that most require this test in a Northeasten City of Brazil

**DOI:** 10.1186/1939-4551-8-S1-A244

**Published:** 2015-04-08

**Authors:** Maria Do Socorro Viana Silva De Sá, Antônia Correia Da Silva

**Affiliations:** 1 Faculdade de Ciências Médicas de Campina Grande, Campina Grande, Paraiba, 58400-250, Brazil; 2 Faculdade de Ciências Médicas de Campina Grande, Maceió, Alagoas, 57055-667,Brazil

## Background

The measurement of total serum IgE is made in some cases to investigate if there is the occurrence of allergic processes. However, does not identify specific antibodies. When it is high may indicate an allergic process, or the ability to initiate some. However, increased IgE levels may also indicate other problems such as parasitic diseases, IgE myeloma, hyper-IgE syndrome and some stages of HIV infection. Therefore, to make a diagnosis of allergy is interesting to use other allergy tests, such as specific IgE (RAST) or prick-test. We surveyed data from patients who underwent the examination of serum total IgE to know which allergic diseases that most affect the examination, which gender and age group most affected, and also calculated an average of IgE in each pathology or group of pathologies.

## Methods

A retrospective observational study of the period 2010 to 2013 was conducted, we analyzed the key data from patient files of an Allergy and Immunology’s public ambulatory service in the city of Campina Grande, who had undergone the examination of serum total IgE.

## Results

Among the 100% investigated (n = 100), 52% were male and 48% female. 61% of range 0-10 years, 10% of 10-20 years old, 13% of 20-30 years old, 9% of 30-40 years old, 3% of 40-50 years old, 4% of 50-60 year old. As pathologies, 27% had allergic rhinitis averaging 379.33 kU / L of total IgE, 18% had Bronchial Asthma averaging 223.89 kU / L of IgE, 11% had the association Allergic Rhinitis and Bronchial Asthma averaging 453.85 KU / L of total IgE, 8% had Atopic Dermatitis with an average of 931.6 KU / L of IgE, 9% had Food Allergy averaging 199.89 KU / L of IgE, and 27% other diseases or associations.

## Conclusion

We conclude that in this service there is an equivalence between the number of men and women who have changes in examination of IgE. We have also seen that children and adolescents are the most feature changes and perform the test. Furthermore, we showed that the majority of patients with high IgE levels has allergic rhinitis, although the values are higher in patients with atopic dermatitis.

